# AMH decline during chemotherapy reflects breast cancer cell DNA damage response (DDR) proficiency: the ONCO AMH1 pilot study

**DOI:** 10.1007/s10815-025-03475-9

**Published:** 2025-04-12

**Authors:** Christine Decanter, Audrey Dassonneville, Emmanuelle D’Orazio, Hélène Behal, Anne-Laure Gagez, Audrey Mailliez, Pascal Pigny

**Affiliations:** 1 Service d’Assistance Médicale à la Procréation et de Préservation de Fertilité, CHU Lille, 59037 Lille, France; 2 Univ. Lille, CANTHER, Inserm UMR 1277, 59045 Lille, France; 3 Service de Biochimie Hormonologie-Métabolisme-Nutrition-Oncologie, CHU Lille, 59037 Lille, France; 4 Service de Biostatistiques, CHU Lille, 59000 Lille, France; 5 Cellule Promotion, DRCI, Centre Oscar Lambret, BRT, 59020 Lille, France; 6 Comité de Sénologie, Centre Oscar Lambret, Pôle d’Oncologie Médicale, 59020 Lille, France

**Keywords:** AMH, Breast cancer, DNA damage response, *BRCA1/2* germline pathogenic variant, Chemotherapy

## Abstract

**Purpose:**

The impact of a germline *BRCA1/2* pathogenic variant (gBRCApv) on baseline or late post-treatment AMH concentrations in breast cancer patients has been extensively studied, yielding mixed conclusions. However, whether the AMH decline during neo-adjuvant chemotherapy reflects differences in chemotherapy susceptibility between gBRCApv carriers and non-carriers remains unexplored.

**Methods:**

A monocentric, retrospective, longitudinal study was conducted on breast cancer patients carrying a gBRCApv (*n* = 12) or wild-type (WT) (*n* = 35) who received a neo-adjuvant sequential chemotherapy (CT) with anthracyclines followed by taxanes. Serum AMH levels were measured at baseline (AMH0) and at three time points during CT by a hypersensitive assay. Tumor size change was assessed via imaging. The impact of genetic status on AMH decline was evaluated using a linear mixed model with post hoc analysis.

**Results:**

The change of AMH concentrations from baseline to the end of CT tended to be influenced by the genetic status (*BRCA* * time interaction, *p* = 0.058). The slope between AMH0 and the end of anthracyclines (after log transformation) was steeper in gBRCApv than in WT patients (mean (SE): − 5.54 (0.63) vs − 3.97 (0.62); *p* = 0.023). Tumor size change was positively and significantly correlated with the change in AMH levels (AMH MidCT-AMH0) in gBRCApv patients (*r* = 0.93, *p* < 0.001) but not in WT patients (*r* = − 0.05; *p* = 0.84).

**Conclusion:**

Germline *BRCA1/2* status influences AMH decline during neo-adjuvant CT with drugs inducing DNA lesions. AMH decay is positively related to tumor size change assessed by imaging in gBRCApv patients. However, no conclusions can be drawn regarding the relationship with treatment response assessed by histological criteria.

## Introduction

Ovarian toxicity is a common adverse effect of most anti-cancer treatments and important to consider because of its negative impact on future fertility of patients [1]. Whether female cancer survivors carrying a genetic deficiency in member(s) of the DNA damage response (DDR) system such as *BRCA1* or *BRCA2* are at higher risk of ovarian toxicity following treatment is currently being questioned [2]. From a biological point of view, the rationale for this hypothesis is based on the demonstrated role of BRCA1/2 in the repair by homologous recombination (HR) [3] of DNA double-strand breaks (DSB) occurring spontaneously in human oocytes [4] and, more generally, in the control of mouse [5] and human ovarian aging [4–7]. Indeed, *BRCA1* mutant heterozygote mice had a lower number of primordial follicles and accumulated DSB in the remaining follicles while aging [5].

However, clinical trials comparing the pool of growing ovarian follicles assessed by serum AMH measurement in patients with and without germline *BRCA1/2* pathogenic variant (gBRCApv) have been inconclusive. For example, in the Turan meta-analysis [8], when AMH is measured at baseline, before chemotherapy (CT), gBRCApv patients have 25% lower concentrations than WT patients. In contrast, two other studies using different assay methods reported no significant difference between these two groups of patients [9, 10]. When AMH was measured at distance from CT, gBRCApv patients showed less recovery (relative to baseline) than WT at 1 year after cessation of treatment [11]. Similarly, germline *BRCA1/2* status is also a predictor of amenorrhea at 12, 18, or 36 months after the completion of chemotherapy [12]. On the other hand, in a study in which AMH was measured longitudinally (before, during, and up to 2 years after completion of CT), the probability of having undetectable AMH levels at 1 or 2 years after CT did not differ between gBRCApv and WT patients [13]. Several factors may explain these discrepancies. First, in all these clinical studies, AMH was measured using conventional assays that have a limit of quantitation (LoQ) that is too high [14] to provide accurate measurements in the expected range of low AMH levels. Second, age at treatment may also influence the results of these studies. Finally, various individual susceptibilities to CT may also impact longitudinal AMH levels change in young breast cancer women [15].

In this pilot study, we aimed to re-examine the hypothesis of a higher risk of ovarian toxicity in gBRCApv patients by focusing on the dynamics of serum AMH during neo-adjuvant chemotherapy (NAC). To achieve this, we analyzed AMH slopes in breast cancer patients with a gBRCApv and WT using a hypersensitive AMH assay [16]. Given that defects in *BRCA1/2* sensitize breast cancer cells to several DNA-damaging agents [17], we also investigated the correlation between AMH decline during NAC and tumor response to treatment in both gBRCApv and WT patients.

## Materials and methods

### Patients

ONCOAMH1 is a retrospective study included young patients (ages 18–38 years) diagnosed with breast cancer, and undergoing neo-adjuvant CT (NAC) with a longitudinal follow up of their ovarian function. This was assessed both clinically (onset and duration of amenorrhea) and biologically by measuring serum AMH levels. These patients were previously enrolled in the prospective KSF1 study (Cancer et Fertilité, NCT 01614704) [13] or were selected from institutional database and had available serum samples in our biobank. All patients underwent genetic testing for *BRCA1/2* germline mutation.

All patients included in ONCOAMH1 had received NAC according to standard protocols. CT regimen included three to four anthracycline-based courses followed by three to four taxane-based courses. The first sequence consisted of epirubicin and cyclophosphamide (three cycles every 3 weeks or four cycles every 2 weeks) resulting in a total dose of epirubicin between 300 and 360 mg/m2 and cyclophosphamide between 1500 and 2400 mg/m2. The second sequence included three cycles of docetaxel (100 mg/m2 every 3 weeks) or 9 to 12 weekly cycles of paclitaxel (80 mg/m2). Patients with HER2-positive tumor received 18 courses of adjuvant trastuzumab for 12 months (3-weekly injections), starting concurrently with the taxanes. None of the patients received GnRH agonist during CT, and no platinum agents were used in the NAC protocol. A total of 47 patients were eligible for the study: 12 with a gBRCApv and 35 WT patients for whom serum samples were available in our biobank. The time points studied were AMH0 (before CT), C2 (day1 of cycle 2 of the anthracycline regimen), Mid-CT (day 1 of the taxane regimen, midway through CT), and End-CT (day 1 of the last CT cycle). Oncological data were retrieved from the patients’ medical files. The Commission for Clinical Studies of the Centre Oscar Lambret has approved the study under the reference CEC- 2023–022. The study complies with the Reference Methodology MR004 of the CNIL (Commission Nationale de l’Informatique & des Libertés). We have checked, before inclusion, that no patient objected to the use of their medical data or samples for research purposes.

### Hormonal investigations

AMH was measured on serum samples using a hypersensitive assay (picoAMH, AnshLabs, Tx USA) as previously described [16, 18]. The assay limit of quantification is 0.023 pmol/L; the assay range is 0.023–7.47 pmol/L for undiluted samples.

### Imaging

All patients underwent initial assessment via breast ultrasound and mammography. The same evaluation was performed at the end of CT, prior to surgery. The change in tumor size after NAC was calculated by comparing the size from the initial (pre-treatment) assessment with the preoperative assessment. Tumor response was evaluated according to the RECIST criteria.

### Pathologic responses

Pathological response was assessed on surgical specimen using the Chevallier classification [19], which was available for all patients. This classification is defined as follows:

- Chevallier 1: no microscopic invasive or in situ carcinoma, and no axillary lymph node metastases
- Chevallier 2: microscopic in situ carcinoma without invasive carcinoma or axillary lymph node metastases
- Chevallier 3: invasive carcinoma with fibrosis or sclerosis
- Chevallier 4: no modification of initial tumor

Chevallier 1 and 2 are considered to reflect a pathological complete response (pCR).

### Statistical analysis

Continuous variables were expressed as median and interquartile ranges (IQR) and compared between gBRCApv and WT patients by Mann–Whitney *U* test. Categorical variables were expressed as counts (*n*) and percentages (%) and compared using *χ*^2^ test or Fisher’s exact test, as appropriate. Changes in AMH levels (after applying a log-transformation) between gBRCApv and WT patients were analyzed using a linear mixed model (an unstructured covariance pattern model) to account for within-patient correlation across repeated measures. The model included time (as a categorical variable), *BRCA1/2* status, and an interaction term (*BRCA* × time) as fixed effects. Post hoc comparisons between gBRCApv and WT patients (changes from baseline to Mid-CT and End-CT time points) were performed using linear contrasts. The association between tumor volume change and AMH level change from baseline to Mid-CT was evaluated by Spearman’s correlation coefficient, separately for each group. Differences in AMH level change from baseline to Mid-CT between Chevallier classification groups [19] were assessed using the Mann–Whitney *U* test. All statistical tests were two-tailed with an alpha risk of 0.05. Statistical analyses were conducted using the SAS software (SAS Institute, version 9.4).

## Results

### Characteristics of breast cancer patients

Twelve patients with a gBRCApv and 35 WT patients with breast cancer were included in the current study. The two groups did not differ in terms of age or weight before CT (Table 1). Tumor size and stage were also comparable. The frequency of lymph node metastasis tended to be higher in gBRCApv than in WT patients (67% vs 34%, *p* = 0.0503). As expected, the pattern of hormone receptor and HER- 2 expression assessed by immunohistochemistry differed significantly between the groups, with a majority of triple negative tumors among gBRCApv patients (92% vs 17%, *p* < 0.001). The patient’s response to NAC, assessed by imaging or pathological examination of the surgically resected tumor, was also similar between the two groups. At the time of data collection, eight (17%) of the patients has relapsed and four(8.5%) had died.

**Table 1:** Clinical characteristics of the study population

	*g BRCA pv* patients (*n* = 12)	*wt* patients (*n* = 35)	*p* value
Age (years)	31 [28–33]	32 [29–33]	0.68
Weight (kg)	69 [63–68]	69 [58–76]	0.73
Tumor classification at diagnosis			
T1	0 (0)	2 (6)	NA
T2	9 (75]	24 (68)	
T3	2 (17)	7 (20)	
T4	1 (8)	2 (6)	
Lymph node status at diagnosis			
N0	4 (33)	23 (66)	0.050
N +	8 (67)	12 (34)	
Immunohistochemical characteristics at diagnosis			
RH + HER2-	1 (8)	9 (26)	< 0.0001^c^
RH − HER2 +	0 (0)	7 (20)	
RH + HER2 +	0 (0)	13 (37)	
Triple negative	11 (92)	6 (17)	
Tumor size at diagnosis (mm)	35 [25–57]	34 [23.2–51.5]	0.94
Tumor response post chemotherapy^a^			
Complete response	2 (18)	11 (31)	NA
Partial response	8 (73)	23 (66)	
Stable disease	1 (9)	1 (3)	
Chevallier classification post-surgery^b^			
Complete response	5 (42)	8 (24)	NA
In situ carcinoma	0 (0)	4 (12)	
Invasive carcinoma	6 (50)	17 (52)	
No or few changes of tumor	1 (8)	4 (12)	

Values are expressed as median [interquartile range] or frequency (%)

*Abbreviations*: *gBRCApv* germline *BRCA1/2* pathogenic variant, *wt* wild type, *NA* not applicable due to frequencies < 5%

^a^The tumor response for one patient in *gBRCApv* group was unavailable

^b^Chevallier classification post-surgery was unavailable for one patient in the *gBRCApv* group and for two patients in the *wt* group

^c^Triple negative vs non-triple negative

### Change of AMH levels during CT

**Fig. 1:**
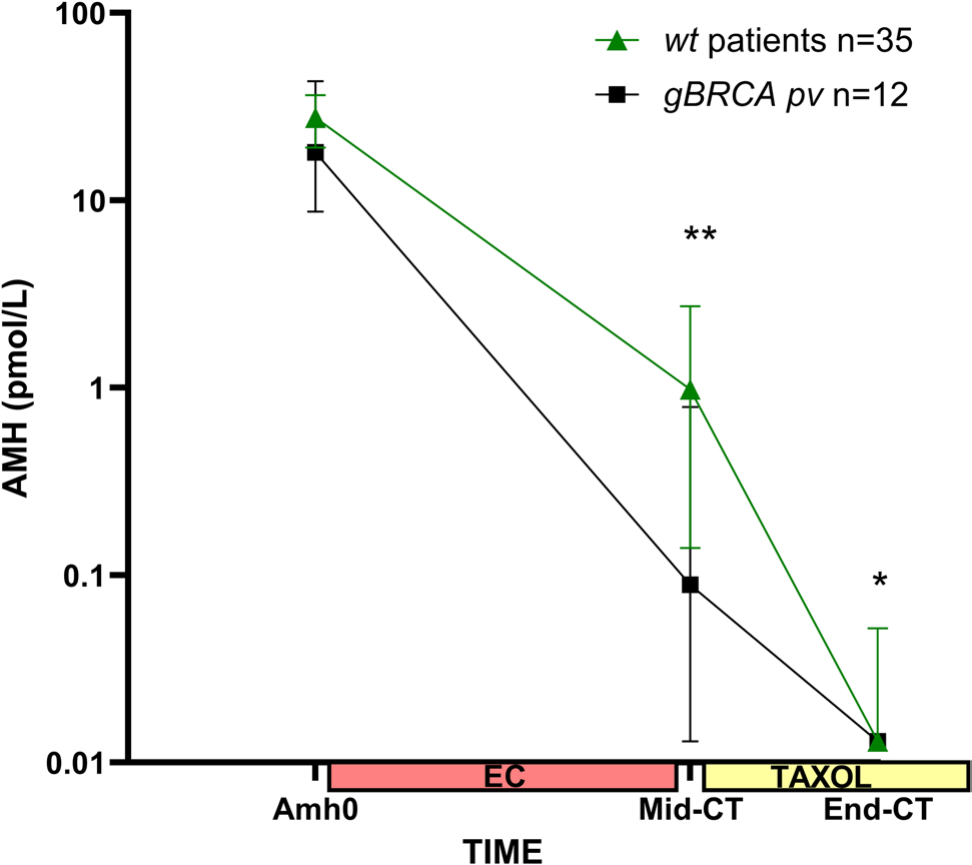
Change in serum AMH levels (expressed as median and 95% CI) between baseline, mid-chemotherapy (Mid-CT), and end of CT (End-CT). **p < 0.01; *p < 0.05 by Mann–Whitney *U* test

AMH concentration was measured before treatment (AMH0) and at three different time points during NAC (C2, Mid-CT, and End-CT). Baseline AMH concentrations were similar between the two groups: 18.06 pmol/L (median) in gBRCApv patients versus 27.6 pmol/L in WT patients (*p* = 0.36). The change in AMH concentrations during NAC, from AMH0 to End-CT, tended to be influenced by the genetic status of the patient (BRCA * time interaction, *p* = 0.058). Post hoc analysis showed that the decline in AMH from AMH0 to Mid-CT was steeper in gBRCApv than in WT (mean in log AMH value (standard error): − 5.54 (0.63) vs − 3.97 (0.62), *p* = 0.024) (Fig. 1). A similar trend was observed for the slope between AMH0 and End-CT: − 7.17 (0.31) in gBRCApv vs − 6.51 (0.24) in WT, *p* = 0.027 (Fig. 1). Despite a visual impression of difference, the slope between Mid-CT and End-CT did not significantly differ between gBRCApv and WT patients: − 1.62 (0.55) vs − 2.53 (0.57), *p* = 0.139.

### AMH decay and oncological outcomes

**Fig. 2:**
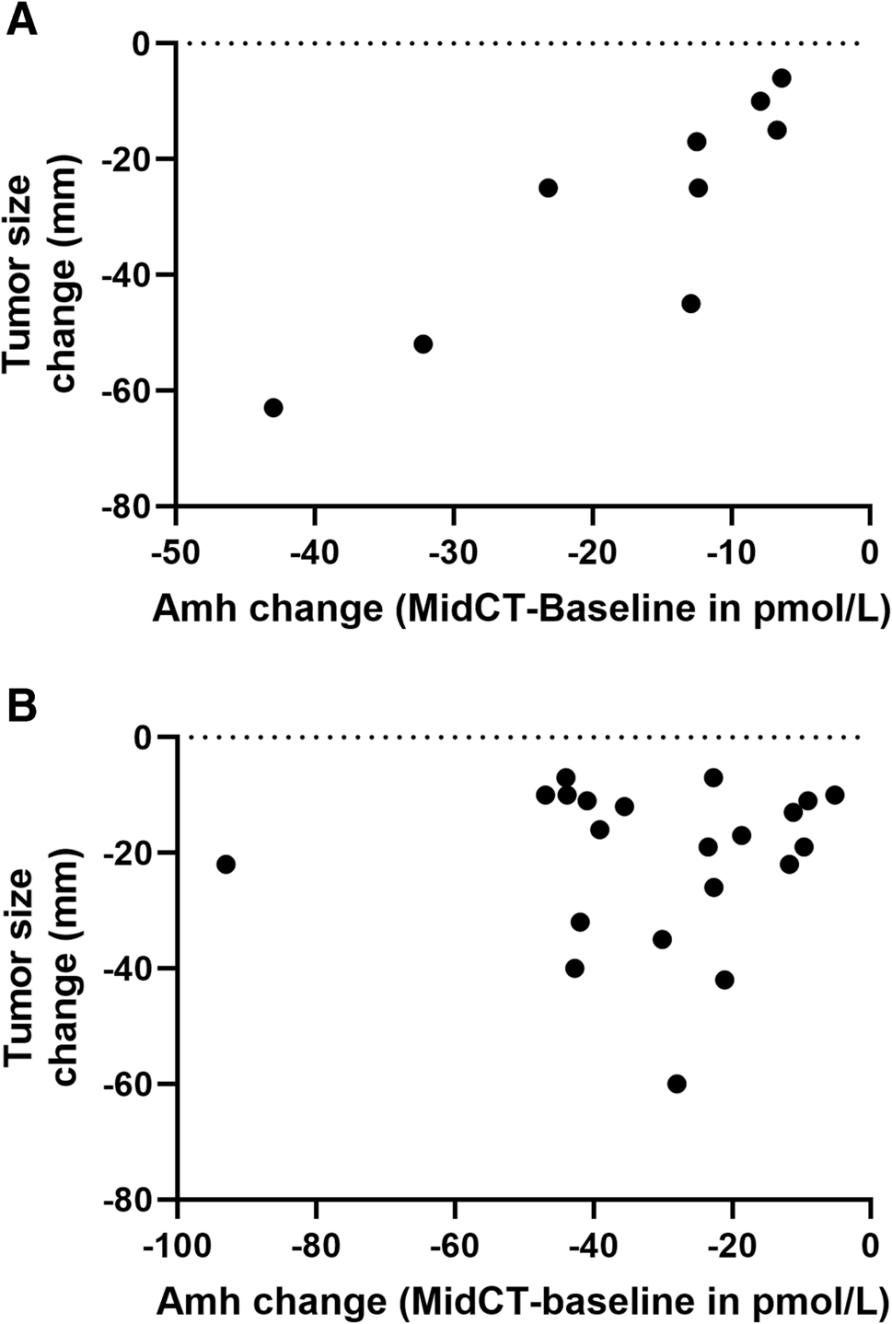
Relationship between tumor size change after chemotherapy (CT) and AMH change (in pmol/l) between baseline and Mid-CT in breast cancer patients with a gBRCApv* (***A**) or wild-type (*wt*) (**B**)

Tumor size change between baseline and End-CT, measured by imaging and expressed as median [IQR] was − 25 [− 45; − 10] mm in gBRCApv and − 17 [− 32; − 11] mm in WT patients, *p* = 0.48. Since 63% of patients had AMH levels below the assay’s LoQ at End-CT, we considered the AMH change over the Mid-CT-AMH0 time frame. As shown in Fig. 2, in gBRCApv patients, tumor size change was positively and significantly correlated with Mid-CT-AMH0 change, *r* = 0.93 (95% CI 0.66–0.98), *p* < 0.001 (Fig. 2A), whereas no correlation was observed in WT patients (*r* = − 0.05 (95% CI − 0.47–0.39), *p* = 0.84 (Fig. 2B).

When tumor response was evaluated post-surgically using the Chevallier classification [19], we observed that the AMH decrease between AMH0 and Mid-CT (expressed in absolute values) was greater in patients with a pCR (Chevallier classes 1 and 2) than in those with residual disease (Chevallier classes 3 and 4) regardless of *gBRCA* status: − 30.10 [− 42.7; − 23.2] pmol/L vs − 12.7 [− 36.9; − 8.8] pmol/L (median [IQR]), *p* = 0.04. However, this difference was not observed when AMH decrease was expressed in relative values: − 94.3% [− 99.3; − 93.4] vs − 97.3% [− 99.9, − 90.8], *p* = 0.87. Moreover, AMH0 was not significantly different between patients with pCR and non pCR cases: 32.5 [21.1–42.4] pmol/L vs 16.2 [11.3–41.5] pmol/L, *p* = 0.088, respectively.

## Discussion

Based on the observation that BRCA1 protein plays a role in controlling ovarian aging through its ability to repair DNA DSBs by HR [3], we aimed to investigate for the first time whether AMH decay curves during NAC differ according to germline *BRCA1/2* genetic status in young breast cancer patients. To date, few studies have explored the gonadotoxic effects of chemotherapy in the context of genetic mutations, with inconclusive results [11, 12, 20]. To accurately measure the expected very low AMH concentrations during NAC, we used a hypersensitive assay previously evaluated [16]. Consistent with previous reports in young breast cancer women [9, 10], baseline AMH concentrations did not differ according to the genetic status. This finding rules out any influence of initial AMH values on the rate of AMH decline as indicated by different mathematical models [21, 22].

Our results highlight a steeper decay of AMH levels in young patients in their thirties with a gBRCApv, compared to WT patients, both between baseline and Mid-CT (at the end of anthracycline treatment) and between baseline and End-CT (at the end of taxanes). A heterozygous deficiency in DNA DSB repair machinery may thus be sufficient to reduce the survival of ovarian growing follicles when facing genotoxic stress induced by gonadotoxic CT. For most patients in both groups, the NAC protocol consisted of three cycles of epirubicin plus cyclophosphamide (EC 100) followed by three cycles of docetaxel. However, some patients received more than six cycles of CT, including four cycles of EC 90 and/or 9 to 12 weekly cycles of paclitaxel instead of the three docetaxel courses. The higher total number of CT cycles in gBRCApv patients should not have influenced the AMH decay curve between AMH0 and Mid-CT, as (i) the cumulative dose of anthracyclins was approximately equivalent in both groups and (ii) the Mid-CT time point corresponds to an assessment at the end of the EC sequence for all patients. Therefore, we can conclude that our data suggest greater gonadal sensitivity in gBRCApv patients to the anthracyclines plus cyclophosphamide regimen. In contrast, the rate of AMH decline between Mid-CT and End-CT did not differ between gBRCApv and WT patients, suggesting similar sensitivity to taxanes in both groups.

From a reproductive perspective, while it remains debated whether fertility in young gBRCApv carriers with a history of breast cancer could be impaired, the risk of premature ovarian aging, compounded by deeper follicular depletion due to CT, along with the indication of ovariectomy to prevent ovarian cancer, supports the systematic recommendation of fertility preservation through oocyte cryopreservation [10, 23].

From an oncological perspective, as BRCA1/2 protein defects sensitize breast tumor cells to various chemotherapeutic agents that induce DNA lesions [17, 24, 25], we investigated whether there was an association between tumor response to NAC and changes in AMH concentrations during NAC in breast cancer patients. Interestingly, we observed a strong positive correlation between tumor size change and AMH levels decrease between baseline and Mid-CT (end of anthracyclines), but this was observed only in the group of gBRCApv patients. How can this selectivity be explained? The *BRCA1/2* pathogenic variant is germline in these patients, so both normal ovarian cells and breast cancer cells exhibit a deficiency in the DNA DSB repair machinery (although the extent of the deficiency likely differs: heterozygous in normal ovarian cells and likely homozygous in breast cancer cells due to a second genetic event, as suggested by Knudson’s hypothesis). Consequently, both cell types should respond similarly to chemotherapeutic drugs that induce DNA DSBs, resulting in DNA damage accumulation, insufficient repair, apoptosis activation, and cell death [26]. In ovarian cells, this leads to reduced AMH biosynthesis. In WT patients, any DNA repair deficit (if present) would only affect breast cancer cells due to somatic *BRCA1/2* mutations, explaining the lack of correlation between AMH decrease and tumor size reduction. This suggests that in gBRCApv patients, ovarian cells mimic the response of breast cancer cells to DNA-damaging chemotherapeutic drugs. Whether this conclusion also applies to other DNA-crosslinking agents, such as platinum salts, used in breast cancer treatment [27] remains to be determined.

From a clinical perspective, these results suggest that, in gBRCApv, monitoring AMH changes during NAC (from baseline to Mid-CT) using a hypersensitive assay could provide early information on the response of BRCA-deficient breast cancer cells to the anthracycline regimen. Additionally, the performance of this new indicator in comparison to other early predictors of tumor response, such as ctDNA [28] or FDG uptake changes [29], remains to be evaluated. However, no conclusions can be drawn regarding the relationship between treatment response, assessed by histological criteria, and AMH changes between baseline and Mid-CT based on the current data. Further studies with larger patient cohorts are necessary.

The strength of this study lies in its first-time investigation of AMH level changes in *BRCA1/2*-mutated versus non-mutated young breast cancer patients, both at baseline and during NAC, alongside tumor size changes. We observed a significant difference between the two populations, suggesting higher gonadotoxicity in the mutated patients. However, we acknowledge that the retrospective design and small size of this pilot study limit the ability to draw firm conclusions. Nevertheless, these findings provide a foundation for future prospective studies with larger populations to better understand the mechanisms of gonadotoxicity and ovarian aging, and to confirm whether tumor response to NAC in gBRCApv patients can be predicted early through serum AMH level changes.

## Data Availability

Upon request to the corresponding author.
